# Radiation Eye Dose for Physicians in CT Fluoroscopy-Guided Biopsy

**DOI:** 10.3390/tomography8010036

**Published:** 2022-02-08

**Authors:** Yohei Inaba, Shin Hitachi, Munenori Watanuki, Koichi Chida

**Affiliations:** 1 Course of Radiological Technology, Health Sciences, Tohoku University Graduate School of Medicine, 2-1 Seiryo, Aoba, Sendai 980-8575, Miyagi, Japan; chida@med.tohoku.ac.jp; 2 Department of Radiation Disaster Medicine, International Research Institute of Disaster Science, Tohoku University, 468-1 Aramaki Aza-Aoba, Aoba, Sendai 980-0845, Miyagi, Japan; 3 Department of Radiology, Tohoku University Hospital, 1-1 Seiryo, Aoba, Sendai 980-8575, Miyagi, Japan; hitachi@rad.med.tohoku.ac.jp; 4 Department of Orthopaedic Surgery, Tohoku University Hospital, 1-1 Seiryo, Aoba, Sendai 980-8575, Miyagi, Japan; munenori.watanuki.e2@tohoku.ac.jp

**Keywords:** CTF-guided biopsy, eye lens, radiation eye dose, radiation protection glasses, scattered radiation

## Abstract

It is important to evaluate the radiation eye dose (3 mm dose equivalent, Hp (3)) received by physicians during computed tomography fluoroscopy (CTF)-guided biopsy, as physicians are close to the source of scattered radiation. In this study, we measured the radiation eye dose in Hp (3) received by one physician during CTF in a timeframe of 18 months using a direct eye dosimeter, the DOSIRIS^TM^. The physician placed eye dosimeters above and under their lead (Pb) eyeglasses. We recorded the occupational radiation dose received using a neck dosimeter, gathered CT dose-related parameters (e.g., CT-fluoroscopic acquisition number, CT-fluoroscopic time, and CT-fluoroscopic mAs), and performed a total of 95 procedures during CTF-guided biopsies. We also estimated the eye dose (Hp (3)) received using neck personal dosimeters and CT dose-related parameters. The physician eye doses (right and left side) received in terms of Hp (3) without the use of Pb eyeglasses for 18 months were 2.25 and 2.06 mSv, respectively. The protective effect of the Pb eyeglasses (0.5 mm Pb) on the right and left sides during CTF procedures was 27.8 and 37.5%, respectively. This study proved the existence of significant correlations between the eye and neck dose measurement (right and left sides, R^2^ = 0.82 and R^2^ = 0.55, respectively) in physicians. In addition, we found significant correlations between CT-related parameters, such as CT-fluoroscopy mAs, and radiation eye doses (right and left sides, R^2^ = 0.50 and R^2^ = 0.52, respectively). The eye dose of Hp (3) received in CTF was underestimated when evaluated using neck dosimeters. Therefore, we suggest that the physician involved in CTF use a direct eye dosimeter such as the DOSIRIS for the accurate evaluation of their eye lens dose.

## 1. Introduction

The International Commission on Radiological Protection (ICRP) adopted the new recommendation of reducing the occupational eye lens dose limit from 150 mSv/year to 20 mSv/year, averaged over 5 years—where the maximum dose should not exceed 50 mSv in any year [1]. The International Atomic Energy Agency (IAEA) has also embraced this new eye lens dose limit for medical workers [2]. Moreover, many countries have used this limit in their regulations. In Japan, the new eye lens dose limit set by the ICRP was adopted in April 2021. Therefore, it is important to evaluate occupational eye doses and eye protection methods [3,4,5].

Computed tomography fluoroscopy (CTF) is one of the main methods used in minimally invasive image-guided procedures for the neck, chest, abdomen, and musculoskeletal system. Additionally, it could be useful to directly assess three-dimensional (3D) data in real time as a guidance tool during various CTF-guided interventions. Thus, CTF-guided biopsy has many advantages with regard to improving performance and procedure times compared to ultrasonography (US) [6,7,8,9,10], and these procedures are being used more frequently. On the other hand, the main disadvantage of CTF-guided interventions is the high radiation exposure of the patient and physician compared with the exposure experienced during conventional CT-guided interventions. During CTF procedures, physicians are situated close to the radiation scattered from patients. Thus, the occupational exposure during CTF is a critical issue for medical staff, especially for the physician involved [11,12,13,14,15,16,17,18]. Many reports have examined the occupational dose received by the eye lenses in various X-ray examinations such as interventional radiology (IR) procedures [19,20,21,22,23,24,25,26,27,28,29,30,31,32,33,34,35,36,37,38,39,40]. However, few studies have discussed the radiation eye dose at 3 mm dose equivalent (Hp (3)) experienced by physicians during CTF-guided biopsy, although there has been a phantom study [41].

Therefore, this study aimed to clarify the occupational eye dose received during CTF-guided biopsies using a direct eye dosimeter. The eye dose measured using direct eye dosimeters was compared to the eye dose estimated using neck dosimeters. Furthermore, we used direct eye dosimeters to evaluate whether lead (Pb) eyeglasses adequately protected the eyes of physicians performing CTF procedures.

## 2. Materials and Methods

### 2.1. Subjects

**Figure 1:**
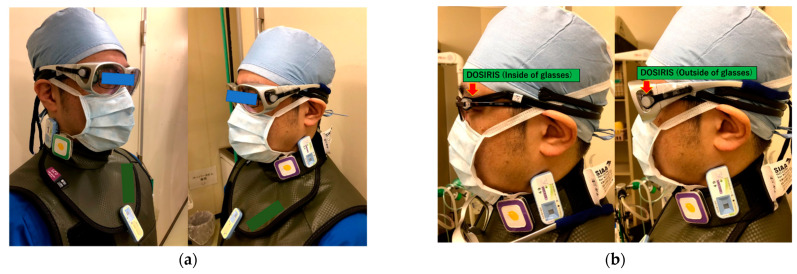
(**a**) The physician wore a protective apron (0.35 mm Pb equivalent), neck guard (0.25 mm Pb equivalent), and Pb glasses during CTF, as well as (**b**) eye dosimeters (DOSIRIS) above and under the Pb eyeglasses.

This study was conducted at Tohoku University Hospital (Sendai, Japan) from July 2018 to December 2019 (18 months). One physician performed all CTF procedures on patients undergoing 95 procedures using an IR-CT system (Aquilion LB; Toshiba, Otawara, Japan). During these CTF procedures, the physician always wore a protective apron (0.35 mm Pb equivalent), neck guard (0.25 mm Pb equivalent), and Pb eyeglasses (0.5 mm Pb equivalent), as shown in Figure 1a. The X-ray conditions in CT-fluoroscopy were a 120 kV-p tube voltage, 20 mA tube current (median value), 0.5 sec rotation time, and 4 mm or 2 mm slice thickness × 3 cross-sections. During CTF-guided biopsy, the physician was positioned close to the right or left side of the patient.

### 2.2. Dosimetry

For the measurement of the radiation eye dose in terms of Hp (3) received by the physician during CTF for 18 months, we used the Hp (3) direct eye dosimeter, the DOSIRIS^TM^ (IRSN, France). The eye dosimeter (DOSIRIS) consisted of a thermoluminescent sensor (^7^LiF: Mg, Ti). We conducted the radiation dose reading of the eye dosimeter every month at Chiyoda-Technol. The physician wore eye dosimeters on the lateral side of the right and left eyes. In addition, for the evaluation of the protective effect of Pb eyeglasses, we placed Hp (3) eye dosimeters inside and outside of them (Figure 1b) for 9 months, from April 2019 to December 2019. The protective effect was evaluated using Equation (1):

(1) $$Protective\;effect\;of\;Pb\;eyeglasses\;\left[\%\right]=\left(1-\frac{Hp\;\left(3\right)\;with\;Pb\;eyeglassses}{Hp\;\left(3\right)\;without\;Pb\;eyeglassses}\right)\times 100$$

For the measurement of the neck dose at Hp (3) received by the physician during CTF, we also used a radiophotoluminesence glass personal dosimeter, the Glass Badge (Chiyoda-Technol, Tokyo, Japan). The glass badge consisted of silver-activated phosphate. Dose calibration for the Hp (3) neck dose was performed at Chiyoda-Technol. The physician neck dose received during CTF was monitored outside of the neck guard to estimate the eye dose received using glass badges (right and left), as shown in Figure 1a. Additionally, we evaluated the correlation between the eye dose and neck dose at Hp (3) to determine whether the eye doses were estimated by using neck glass batches. Moreover, we recorded CT dose-related parameters (acquisition numbers, fluoroscopy time, mAs, CT dose index (CTDI), dose length product (DLP), etc.) to estimate the eye dose received from performing 95 consecutive procedures involving CTF-guided biopsy.

### 2.3. Statical Analysis

Liner regression was used to evaluate the correlations between the radiation doses recorded by the neck dosimeter and eye dosimeter, as well as the correlations between the radiation doses recorded by the internal dosimeter and external dosimeter. Determination coefficient analysis was used to evaluate whether the CT dose-related parameters were linearly related to occupational doses (eye and neck doses).

All statistical analysis were performed based on the JMP Pro 16 software (SAS Institute Inc., Cary, NC, USA). We defined the statistical significance as *p* < 0.05.

## 3. Results

Table 1 summarizes the results of our 18-month study on patients undergoing a total of 95 procedures involving CTF-guided biopsies. The averages ± SDs of the CT-fluoroscopic acquisition numbers, CT-fluoroscopic time, and CT-fluoroscopic mAs were 26.2 ± 15.5, 20.0 ± 11.8 s, and 464.1 ± 327.4, respectively. Regarding the standing positions of the physicians performing the 95 CTF procedures, 49 were on the right and 46 were on the left.

### 3.1. Physician Dose

The total physician eye doses (right and left) received in terms of Hp (3) when not wearing Pb glasses for 18 months in CTF procedures (95 procedures) were 2.25 and 2.06 mSv, respectively. Additionally, the total neck doses (right and left) received by physicians in terms of Hp (3) without Pb glasses for 18 months were 1.16 and 1.02 mSv, respectively (Table 2). The neck dose of Hp (3) tended to underestimate the eye dose. The right eye doses for inside and outside the Pb eyeglasses for 9 months (47 procedures) were 0.52 and 0.72 mSv, respectively. Additionally, the left eye doses for the inside and outside of Pb eyeglasses were 0.50 and 0.80 mSv, respectively. Therefore, the protective effect of Pb eyeglasses on the right and left sides was 27.8 and 37.5%, respectively (Table 3).

### 3.2. Relationship between the Eye Dosimeter and Neck Dosimeter or CT-Related Parameters

**Figure 2:**
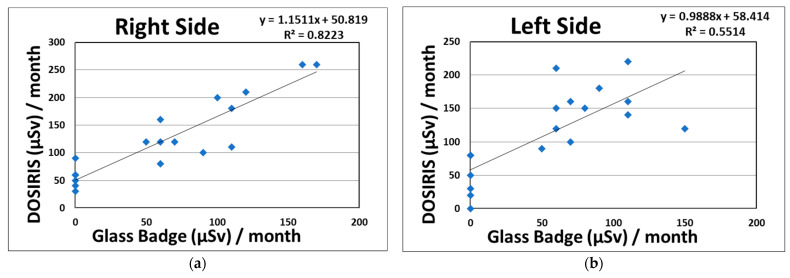
Relationship between the eye dosimeter (DOSIRIS, Hp (3)) and neck dosimeter (Glass Badge, Hp (3)), taken monthly during CTF-guided biopsies (95 procedures). (**a**) Right side dosimeter; (**b**) left side dosimeter.

**Figure 3:**
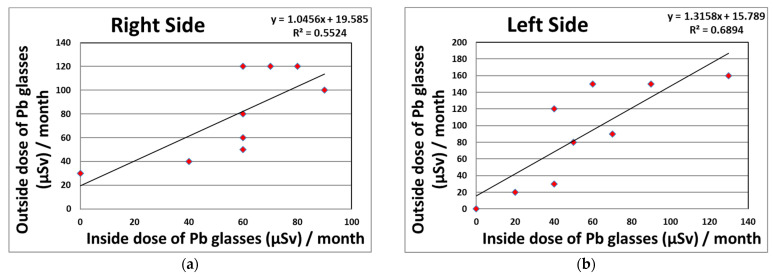
Relationship between the eye dosimeter measurements (DOSIRIS, Hp (3)) inside and outside the Pb eyeglasses, taken monthly during CTF-guided biopsies (47 procedures). (**a**) Right side dosimeter; (**b**) left side dosimeter.

We proved the existence of significant correlations between the eye and neck dosimeter measurements (right side: R^2^ = 0.82; left side: R^2^ = 0.55) monthly in CTF for 95 procedures (Figure 2). Figure 3 shows the correlations between the eye dosimeter measurements (right side: R^2^ = 0.55; left side: R^2^ = 0.69) inside and outside the Pb eyeglasses, taken monthly for 47 procedures involving CTF-guided biopsies.

Table 4 shows the significant correlations between the physician doses (eye and neck) received and the CT dose-related parameters—especially CT-fluoroscopic mAs (right eye dose: R^2^ = 0.50; left eye dose: R^2^ = 0.52; right neck dose: R^2^ = 0.75; and left neck dose: R^2^ = 0.59). Meanwhile, there were no significant correlations between the physician doses and other CT-related parameters, such as CTDI vol. and DLP.

## 4. Discussion

Evaluation of radiation exposure received by patients in medicine is very important [42,43,44,45,46,47,48,49,50,51]. Furthermore, occupational radiation protection in medical workers is a critical problem [52,53,54]. The ICRP suggested that tissue reactions of the eyes (e.g., cataracts) can occur at lower radiation doses than those examined in previous epidemiological research [1]. The IAEA recommends wearing a dosimeter as close to the eye as possible so that the lens dose of the eye can be measured the most accurately [55]. ICRP Publication 103 recommends that Hp (3) should be used to measure the equivalent dose given to the lens of the eye [56]. The largest groups of workers who may be affected by lowering the lens dose limit of the eye are employed in the medical sector and are involved in CTF-guided interventional procedures [55,57]. Therefore, medical workers, during CTF-guided biopsies, should be to assess the eye dose and eye protection [6,7,8,9,10,11,12,13,14,15,16,17,18,41,58].

In this study, we revealed that the total eye doses on the right and left sides in terms of Hp (3) received by a physician without Pb eyeglasses for 18 months during CTF procedures were 2.25 and 2.06 mSv, respectively. It was expected that the eye dose limit (20 mGy/year) would not be exceeded. Regarding the total neck doses (right and left), the physician doses were 1.16 and 1.02 mSv, respectively. As shown in Table 2 and Figure 2, the radiation eye dose received by physicians involved in CTF procedures was underestimated by approximately two-fold when using neck badge measurements compared with the direct eye dosimeter measurements carried out using DOSIRIS, although the correlation between the eye and neck dosimeter measurements was high (right side: R^2^ = 0.82; left sides: R^2^ = 0.55). This may have been because the angular dependences of the DOSIRIS show better dose responses at almost all angles than that of the neck badge [59]. Hence, we suggest that for the direct evaluation of eye doses received by physicians, an eye dosimeter such as the DOSIRIS should be used. In addition, physicians attending the CTF are advised to place the direct dosimeter as close as possible to the eyes in order to assess accurate radiation doses.

As shown in Table 3 and Figure 3, the protective effects of Pb eyeglasses on the right and left sides were 27.8 and 37.5%, respectively. Using protective eyeglasses can further reduce the lens dose received by the physician. However, several studies carried out during IR have reported that the protection provided by radiation protective eyeglasses in clinical settings was approximately 50–60% [24,27,31]. In our results, we found lower effects than those seen in other studies, although the eye dosimeter measurements (DOSIRIS, Hp (3)) clarified the correlations between the inside and outside of the Pb eyeglasses of 0.5 mm Pb equivalent (right side: R^2^ = 0.55; left sides: R^2^ = 0.69), taken monthly for 47 CTF procedures. This may have been because the distribution of scattered radiation in CTF is almost the same in the height direction regardless of the protection used [58].

As shown in Table 4, we showed the existence of significant correlations between the physician doses (eye and neck dose) and CT dose-related parameters—especially CT-fluoroscopic mAs (right eye dose: R^2^ = 0.50; left eye dose: R^2^ = 0.52; right neck dose: R^2^ = 0.75; and left neck dose: R^2^ = 0.59). Thus, we recommend minimizing the CT-fluoroscopic acquisition numbers and lowering the CT-fluoroscopic mAs to reduce the physician doses. In addition, it could be useful to estimate physician doses from CT dose-related parameters such as CT fluoroscopic mAs.

In summary, our study measured the occupational eye doses at Hp (3) received by physicians working in CTF procedures for 18 months. We evaluated the protective effect of Pb eyeglasses with 0.5 mm Pb equivalent. We also estimated the radiation eye doses received by physicians using personal neck dosimeters. The eye lens doses recorded during CTF using neck dosimeters were underestimated by approximately two-fold compared with direct eye dosimeter measurements carried out using the DOSIRIS. Therefore, we suggest that physicians involved in CTF use a direct eye dosimeter (e.g., DOSIRIS) to accurately assess the lens dose of the eye.

Our study has some limitations. This study evaluated the physician doses only at our institution using the same CT. For the CT-fluoroscopic X-ray conditions, we used our hospital protocol. In addition, all CTF procedures were performed by one physician. Therefore, our results might be different for physicians who require different lengths of time to complete procedures, as well as those who need to be at a longer or shorter distance from the intervention site.

## 5. Conclusions

This study investigated the eye doses associated with CTF-guided biopsies received by physicians using a direct eye dosimeter at our hospital. To reduce the eye dose, we recommend that the physician wear Pb eyeglasses. The eye dose in terms of Hp (3) received in CTF was underestimated when evaluated using neck dosimeters. Therefore, to correctly assess the eye dose at Hp (3), it is essential to use direct eye dosimeters such as DOSIRIS.

## Figures and Tables

**Table 1:** Summary of our 18-month study on patients undergoing CTF-guided biopsies (95 procedures).

Characteristics	Ave. ± SD	Median	Range
Age (years)	51.8 ± 20.8	55.5	5.0–88.0
Body Mass Index	23.2 ± 3.9	22.7	15.8–34.3
CT-fluoroscopic acquisition number	26.2 ± 15.5	22.0	8.0–96.0
CT-fluoroscopic time (s)	20.0 ± 11.8	17.1	5.9–72.1
CT-fluoroscopic mAs	464.1 ± 327.4	376.0	118.0–2105.0
CTDI vol (mGy)	10.2 ± 5.3	7.7	3.3–29.6
DLP (mGy*cm)	215.1 ± 88.5	186.7	76.2–539.9
Target depth (mm)	60.5 ± 21.6	56.5	14.3–119.5

Ave.: average; SD: standard deviation; CTDI: CT dose index; DLP: dose length product.

**Table 2:** Total physician doses (eye and neck) received in 18 months during CTF procedures (95 procedures).

(μSv)	Right Side	Left Side
Eye dose (DOSIRIS), Hp (3)	2250	2060
Neck dose (Glass Badge), Hp (3)	1160	1020

**Table 3:** Protective effect of Pb eyeglasses used for 9 months during CTF procedures (47 procedures).

(μSv)	Outside of Eyeglasses	Inside of Eyeglasses	Protective Effect (%)
Right eye dose (DOSIRIS), Hp (3)	720	520	27.8
Left eye dose (DOSIRIS), Hp (3)	800	500	37.5

**Table 4:** Determination coefficient (R^2^) between physician doses and CT dose-related parameters (95 procedures).

Determination Coefficient (R^2^)	CT-Acquisitions No.	CT-Fluoroscopic Time (s)	CT-Fluoroscopic mAs
Right eye dose (DOSIRIS), Hp (3)	0.463	0.441	0.497
Left eye dose (DOSIRIS), Hp (3)	0.507	0.486	0.524
Right neck dose (Glass Badge), Hp (3)	0.788	0.799	0.745
Left neck dose (Glass Badge), Hp (3)	0.666	0.675	0.592

## Data Availability

Not Applicable.
